# The Differential DRP1 Phosphorylation and Mitochondrial Dynamics in the Regional Specific Astroglial Death Induced by Status Epilepticus

**DOI:** 10.3389/fncel.2016.00124

**Published:** 2016-05-18

**Authors:** Ah-Reum Ko, Hye-Won Hyun, Su-Ji Min, Ji-Eun Kim

**Affiliations:** Department of Anatomy and Neurobiology, Institute of Epilepsy Research, College of Medicine, Hallym UniversityChuncheon, South Korea

**Keywords:** astroglial death, clasmatodendrosis, DRP1, mitochondria, status epilepticus

## Abstract

The response and susceptibility to astroglial degenerations are relevant to the distinctive properties of astrocytes in a hemodynamic-independent manner following status epilepticus (SE). Since impaired mitochondrial fission plays an important role in mitosis, apoptosis and programmed necrosis, we investigated whether the unique pattern of mitochondrial dynamics is involved in the characteristics of astroglial death induced by SE. In the present study, SE induced astroglial apoptosis in the molecular layer of the dentate gyrus, accompanied by decreased mitochondrial length. In contrast, clasmatodendritic (autophagic) astrocytes in the CA1 region showed mitochondrial elongation induced by SE. Mdivi-1 (an inhibitor of mitochondrial fission) effectively attenuated astroglial apoptosis, but WY14643 (an enhancer of mitochondrial fission) aggravated it. In addition, Mdivi-1 accelerated clasmatodendritic changes in astrocytes. These regional specific mitochondrial dynamics in astrocytes were closely correlated with dynamin-related protein 1 (DRP1; a mitochondrial fission protein) phosphorylation, not optic atrophy 1 (OPA1; a mitochondrial fusion protein) expression. To the best of our knowledge, the present data demonstrate for the first time the novel role of DRP1-mediated mitochondrial fission in astroglial loss. Thus, the present findings suggest that the differential astroglial mitochondrial dynamics may participate in the distinct characteristics of astroglial death induced by SE.

## Introduction

Mitochondria are morphologically dynamic organelles that are essential for maintaining cell function, growth, and survival (MacAskill et al., 2010; Sheng and Cai, 2012; Birsa et al., 2013). The morphological state of mitochondria is linked to their energy production capacity (Bach et al., 2003; Olichon et al., 2003; Chen et al., 2005; Benard and Rossignol, 2008) and cell death mechanisms (Cheung et al., 2007; Detmer and Chan, 2007; Jahani-Asl et al., 2007; Chen and Chan, 2009; Rintoul and Reynolds, 2010). Mitochondria undergo two opposing events, fission and fusion (termed mitochondrial dynamics) that operate in equilibrium to form small individual units or interconnected networks. These processes play critical roles in mitochondrial functions and activity-dependent regulation of mitochondrial distribution in various cells (Li et al., 2004; Sung et al., 2008). Mitochondrial dynamics are regulated by GTPases, an evolutionary conserved large family of proteins (Chen and Chan, 2005). The fusion-associated GTPases, mitofusin 1 (MFN1), MFN2 and optic atrophy 1 (OPA1) increase networks of elongated mitochondria (Chen et al., 2003; Rambold et al., 2011). In contrast, the mitochondrial fission protein dynamin-related protein 1 (DRP1) produces small punctuated mitochondria by interacting with fission related protein-1 (Fis-1) and mitochondrial fission factor (MFF; Smirnova et al., 2001; Chen et al., 2003; Cipolat et al., 2004). Among them, DRP1 is essential for most types of mitochondrial fission. The regulation of DRP1 by post-translational modifications is important for DRP1 translocation to mitochondria (Alaimo et al., 2014). Phosphorylation of DRP1 at Ser 616 (S616) by cyclin dependent kinase (CDK) 1/Cyclin B or CDK5 promotes mitochondrial fission during mitosis (Taguchi et al., 2007; Liesa et al., 2009). In contrast, DRP1-S637 phosphorylation by protein kinase A (PKA) induces the detachment of DRP1 from mitochondria and inhibits mitochondrial fission (Kashatus et al., 2011; Wang et al., 2012). Thus, dephosphorylation of DRP1-S637 by calcineurin facilitates its translocation to mitochondria and subsequently increases mitochondrial fission, which leads to an increased response to apoptotic stimuli (Campello and Scorrano, 2010). Therefore, imbalance of DRP1 S616/S637 phosphorylation ratio is involved in pathogenesis of neurological diseases (DuBoff et al., 2012; Kim et al., 2014b).

Although astrocytes have been thought as simple supporting cells to have homogenous properties, a number of studies demonstrate that properties in astrocytes are distinct in different brain regions. Indeed, the regional specific patterns of astroglial death are reported before or after neuronal damage or reactive astrogliosis (Borges et al., 2006; Kang et al., 2006; Kim et al., 2014a). In particular, status epilepticus (SE; prolonged seizure activity) leads to astroglial death in the rat hippocampus and piriform cortex (PC), which shows regional specific patterns (Kang et al., 2006; Kim et al., 2010a,b, 2011, 2014a; Ryu et al., 2011a,b). Astroglial apoptosis is observed in the molecular layer (not the hilus) of the dentate gyrus (Kang et al., 2006; Kim et al., 2008, 2010b). In the CA1 region, clasmatodendrosis is a typical irreversible astroglial degeneration via excessive lysosome-derived autophagy representing vacuolization of edematous cell bodies and disintegrated processes (Penfield, 1928; Sugawara et al., 2002; Kim et al., 2008, 2010b; Ryu et al., 2011a,b). In the PC, focal astroglial necrosis is observed (Kim et al., 2013, 2014a) similar to the various brain regions (Ingvar et al., 1994; Schmidt-Kastner and Ingvar, 1994; Gualtieri et al., 2012). Furthermore, we have recently reported that these differential response and susceptibility to astroglial degenerations are most likely due to the distinctive properties of astrocytes in a hemodynamic-independent manner (Kim et al., 2014a). Since DRP1 phosphorylation plays an important role in mitosis, apoptosis and programmed necrosis (Taguchi et al., 2007; Liesa et al., 2009; Campello and Scorrano, 2010; Kashatus et al., 2011; DuBoff et al., 2012; Wang et al., 2012; Kim et al., 2014b), it is likely that the differential pDRP1-S616 or -S637 level would be involved in these regional specific astroglial degenerations induced by SE. In the present study, therefore, we investigated whether the unique pattern of DRP1 phosphorylation is closely relevant to the characteristics of SE-induced astroglial death, and the modulation of mitochondrial dynamics affects the regional specific astroglial degeneration in response to SE.

## Materials and Methods

### Experimental Animals and Chemicals

Male Sprague-Dawley rats (7 weeks old) were obtained from Daehan Biolink (South Korea). The animals were provided with a commercial diet and water *ad libitum* under 22 ± 2°C, 55 ± 5% and a 12:12 light/dark cycle conditions. Animal protocols were approved by the Institutional Animal Care and Use Committee of Hallym University (Chuncheon, South Korea). All reagents were obtained from Sigma-Aldrich (St. Louis, MO, USA), except as noted.

### Surgery and Drug Infusion

Rats were anesthetized with 1–2% Isoflurane in O_2_ and placed in a stereotaxic frame. A brain infusion kit 1 (Alzet, USA) was implanted into the right lateral ventricle (1 mm posterior; 1.5 mm lateral; 3.5 mm depth), and connected to an osmotic pump (1007D, Alzet, USA) containing: (1) vehicle; (2) Mdivi-1 (50 μM); or (3) WY 14643 (150 μM). Mdivi-1 or WY14643 pretreatment did not affect the seizure susceptibility or its vulnerability in response to pilocarpine and animal survival rates following SE (Kim et al., 2014b). The pump was placed in a subcutaneous pocket in the interscapular region.

### SE Induction

Three days after surgery, rats were treated with pilocarpine (380 mg/kg, i.p.). To reduce peripheral effects of pilocarpine, Atropine methylbromide (5 mg/kg, i.p.) was injected 20 min before a single dose of pilocarpine. Animals were maintained in SE for 2 h, after which diazepam (10 mg/kg, i.p.) was administered to terminate seizure activity, and repeated, as needed. After SE, all animals were observed in the small animal intensive care units (DW-1, ThermoCare, Paso Robles, CA, USA) and given 5% dextrose in lactate Ringer solution (5 ml S.C. after fluids are warmed to normal body temperature). To prevent drying of eyes, an ocular lubricant was applied. Animals were continuously monitored and injected with 5% dextrose in lactate Ringer solution at 4 h interval when needed. Next day, animals were fed moistened high-fat rodent chow and apple slices on the floor cage. As controls, age-matched normal rats were treated with saline instead of pilocarpine.

### Tissue Processing and Immunohistochemistry

Under urethane anesthesia (1.5 g/kg, i.p.), rats were transcardially perfused with 4% paraformaldehyde in 0.1 M phosphate buffer (PB, pH 7.4). After postfixation in the same fixative for 4 h, brains were infiltrated with 30% sucrose and sectioned with a cryostat at 30 μm. Sections were incubated overnight at room temperature in a mixture of primary antisera (Table 1) in PBS containing 0.3% Triton X-100, and subsequently in a mixture of FITC- and Cy3-conjugated IgG (Amersham, NJ, USA). TUNEL staining was also applied according to the manufacturer’s protocol (Upstate, Lake Placid, NY, USA). For negative control, the hippocampal tissues obtained from non-SE and post-SE animals were incubated with pre-immune serum instead of primary antibody. Images were captured using an Axiocam HRc camera and AxioVision Rel. 4.8 software or a confocal laser scanning microscope (LSM 710, Carl Zeiss Inc., Oberkocken, Germany). Images of each section on the monitor were captured (15 sections per each animal). After regions were outlined, areas of interest (500 μm^2^/area) were selected from the stratum radiatum of the CA1 field and the molecular layer of the dentate gyrus. Each image was normalized by adjusting the black and white range of the image using AxioVision Rel. 4.8 Software. Fluorescent intensity was then standardized by setting the threshold levels (mean background intensity obtained from 5 image input). Fluorescent intensity values were corrected by subtracting the average values of background noise (threshold level). Manipulation of the images was restricted to threshold and brightness adjustments to the whole image.

**Table 1:** **Primary antibodies used in the present study**.

Antibody	Host	Manufacturer (catalog number)	Dilution used
GFAP	Rabbit	Abcam (ab7260)	1:500
GFAP	Mouse	Millipore (Mab3402)	1:100
LAMP-1	Rabbit	Abcam (ab24170)	1:100
Mitochondrial marker	Mouse	Abcam (ab14705)	1:500
(Mitochondrial complex IV subunit 1, MTCO1)
pDRP1 S616	Rabbit	Cell Signaling (4494)	1:500
pDRP1 S637	Rabbit	Cell Signaling (4867)	1:500

### Cell Count and Measurement of Mitochondrial Length

For quantification of immunohistochemical data, images of each section on the monitor were captured (15 sections per each animal). After regions were outlined, areas of interest (1 × 10^5^ μm^2^) were selected from the stratum radiatum of the CA1 field and the molecular layer of the dentate. Cells were counted on 20× images using AxioVision Rel. 4.8 Software. Results are presented as means ± S.D. Individual mitochondrion length in astrocytes (*n* = 20/section) was also measured by using AxioVision Rel. 4.8 Software or ZEN lite (Blue Edition, Carl Zeiss Inc., Oberkocken, Germany) software following 3D-reconstruction (Figure 2A). Cell counts and measurement of mitochondrial length were performed by two different investigators who were blind to the classification of tissues.

### Data Analysis

All data obtained from the quantitative measurements were analyzed using one-way ANOVA to determine statistical significance. Bonferroni’s test was used for *post hoc* comparisons. A *p*-value below 0.05 was considered statistically significant.

## Results

### Regional Specific Astroglial Death Induced by SE

**Figure 1:**
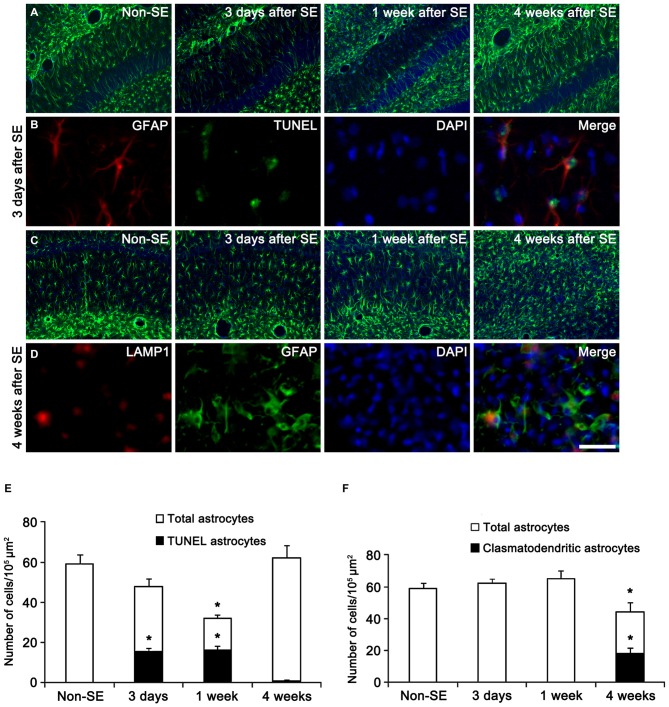
**Regional specific astroglial death in the hippocampus following status epilepticus (SE). (A)** Astroglial responses in the molecular layer of the dentate gyrus. Massive astroglial loss is observed in this region 3 days and 1 week after SE. **(B)** TUNEL-positive apoptosis in the molecular layer of the dentate gyrus 3 days after SE. **(C)** Astroglial responses in the CA1 region following SE. **(D)** LAMP1-positive clasmatodendrosis (round-shaped edematous cell body, short blunt processes, loss of distal processes, GFAP aggregation, nuclear dissolution and LAMP-1 positive vacuolization) in this region 4 weeks after SE. Bar = 100 **(A,C)** and 25 μm **(B,D)**. **(E)** Quantification of the fraction of TUNEL-positive astrocytes in the total astrocytes within the molecular layer of the dentate gyrus following SE (mean ± SD, *n* = 7, respectively). *p < 0.05 vs. non-SE animals. **(F)** Quantification of the fraction of LAMP1-positive astrocytes in the total astrocytes within the CA1 region following SE (mean ± SD, *n* = 7, respectively). *p < 0.05 vs. non-SE animals.

In the present study, all negative controls for immunohistochemistry resulted in the absence of immunoreactivity in any structures (data not shown). First, we confirmed the regional specific vulnerability of astrocytes to SE insults. Consistent with our previous studies (Kang et al., 2006; Kim et al., 2010b, 2011), SE induced massive astroglial apoptosis in the molecular layer of the dentate gyrus in the present study. Briefly, 32 and 51% of astrocytes showed TUNEL signals in this region 3 and 7 days after SE, respectively (Figures 1A,B,E). Four weeks after SE, reactive astrocytes were detected in this region (Figures 1A,B,E). In the CA1 region, typical reactive astrogliosis was observed at 3 and 7 days after SE (Figures 1C,D,F). Four weeks after SE, 13% of CA1 astrocytes showed typical clasmatodendrosis, which had round-shaped edematous cell body, short blunt processes, loss of distal processes, GFAP aggregation, nuclear dissolution (absence of nucleus or watery pale nuclear staining) and LAMP-1 positive lysosomes (Figures 1C,D,F). These findings indicate that SE may lead to the regional specific astroglial death in the hippocampus.

### Regional Specific Mitochondrial Dynamics in Astrocytes Following SE

**Figure 2:**
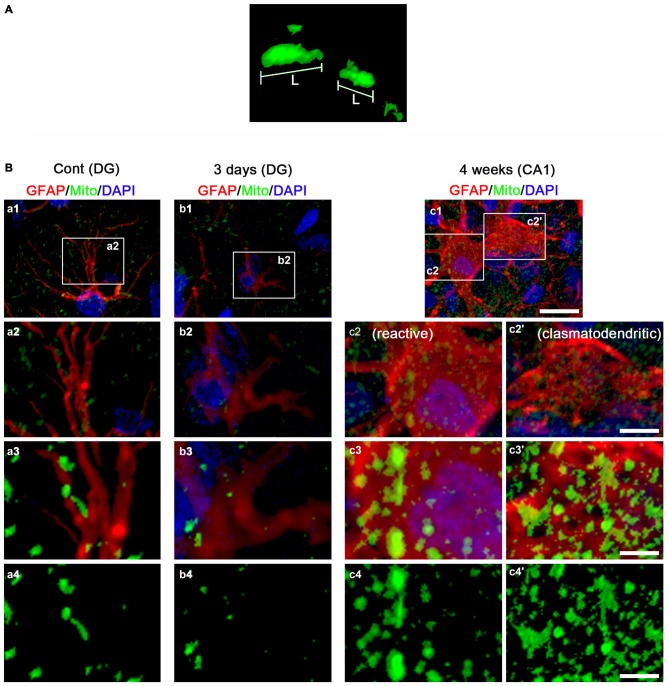
**3D-reconstruction of mitochondria in astrocytes within the hippocampus. (A)** Representative image demonstrating the measurement of mitochondrial length. L, mitochondrial length (long axis). **(B)** Representative photos of 3D-reconstruction of mitochondria in astrocytes within the CA1 region and the dentate gyrus. Panels 1–3 are merge images for GFAP, mitochondrial marker and DAPI. Panel 4 demonstrates only image for mitochondrial marker. Panels c2,3 and c2’,3’ indicate reactive astrocytes and clasmatodendritic astrocytes, respectively. Panels 2,3 are high magnification images for rectangles in panels 1–3. Bar = 30 (panel 1), 7.5 (panel 2) and 3.75 (panels 3,4) μm.

**Figure 3:**
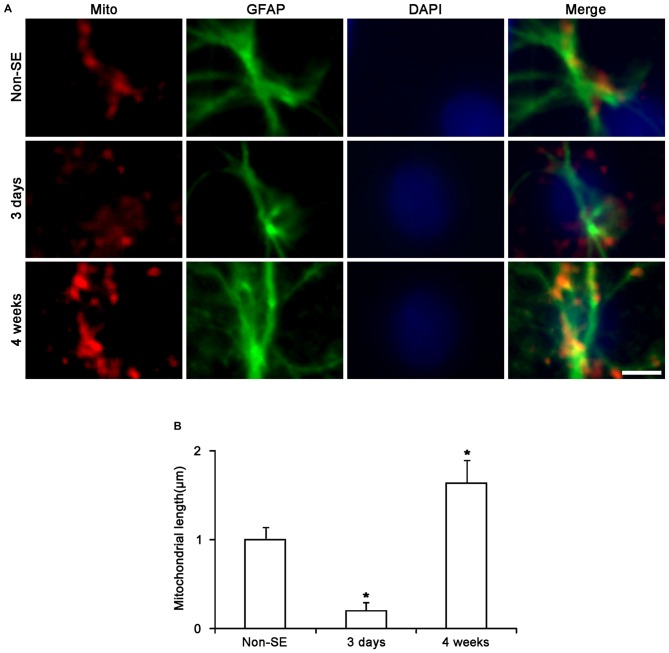
**Changes in mitochondrial morphology in astrocytes within the molecular layer of the dentate gyrus. (A)** As compared to non-SE animals, mitochondrial length is reduced 3 days after SE. Four weeks after SE, mitochondria are elongated and the morphology of mitochondria is sphere shaped in reactive astrocytes. Bar = 3.75 μm. **(B)** Quantitative values (mean ± SEM) of mitochondrial length in the molecular layer of the dentate gyrus (*n* = 7, respectively). *p < 0.05 vs. non-SE animals.

To investigate whether mitochondrial dynamics are relevant to SE-induced astroglial death, we analyzed the mitochondrial length in astrocytes. In the molecular layer of the dentate gyrus of non-SE animals, mitochondrial length was ~1 μm in astrocytes (Figures 2B, 3A,B). Three days after SE, mitochondrial length was reduced to <0.2 μm in astrocytes (*p* < 0.05 vs. non-SE animals, Figures 2B, 3A,B). Four weeks after SE, mitochondrial length was increased to 1.64 μm and the morphology of mitochondria was sphere shaped in reactive astrocytes (sphere formation, *p* < 0.05 vs. non-SE animals, Figures 3A,B).

**Figure 4:**
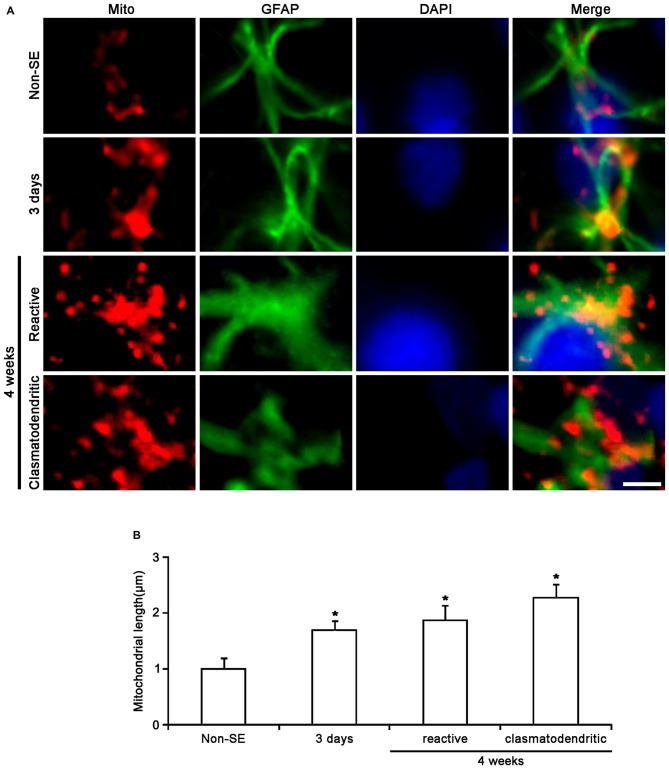
**Change in mitochondrial morphology in astrocytes within the CA1 region. (A)** As compared to non-SE animals, mitochondrial length is increased 3 days after SE. Four weeks after SE, mitochondrial length is increased and the morphology of mitochondria is sphere shaped in reactive astrocytes as well as clasmatodendritic astrocytes. Bar = 3.75 μm. **(B)** Quantitative values (mean ± SEM) of mitochondrial length in the CA1 region (*n* = 7, respectively). *p < 0.05 vs. non-SE animals.

In the CA1 region of non-SE animals, mitochondrial length was ~1 μm in astrocytes (Figures 4A,B). Three days after SE, mitochondria were elongated to 1.69 μm and showed sphere formation in astrocytes (*p* < 0.05 vs. non-SE animals, Figures 4A,B). Four weeks after SE, mitochondrial length (1.87 μm) and sphere formation were increased in reactive astrocytes (*p* < 0.05 vs. non-SE animals, Figures 2B, 4A,B). In clasmatodendritic astrocytes, mitochondrial length was 2.28 μm (*p* < 0.05 vs. non-SE animals, Figures 2B, 4A,B). These findings indicate that the differential patterns of mitochondrial dynamics may be relevant to regional specific astroglial death induced by SE.

### Effects of Mdivi-1 and WY14643 on Regional Specific Astroglial Responses to SE

To directly address the issue of whether the mitochondrial dynamics influence on the regional specific astroglial death in response to SE, we investigated the effect of Mdivi-1 (an inhibitor of mitochondrial fission) and WY14643 (an enhancer of mitochondrial fission; Lundgren et al., 1990; Zolezzi et al., 2013) on astroglial mitochondrial dynamics within the molecular layer of the dentate gyrus and the CA1 region.

**Figure 5:**
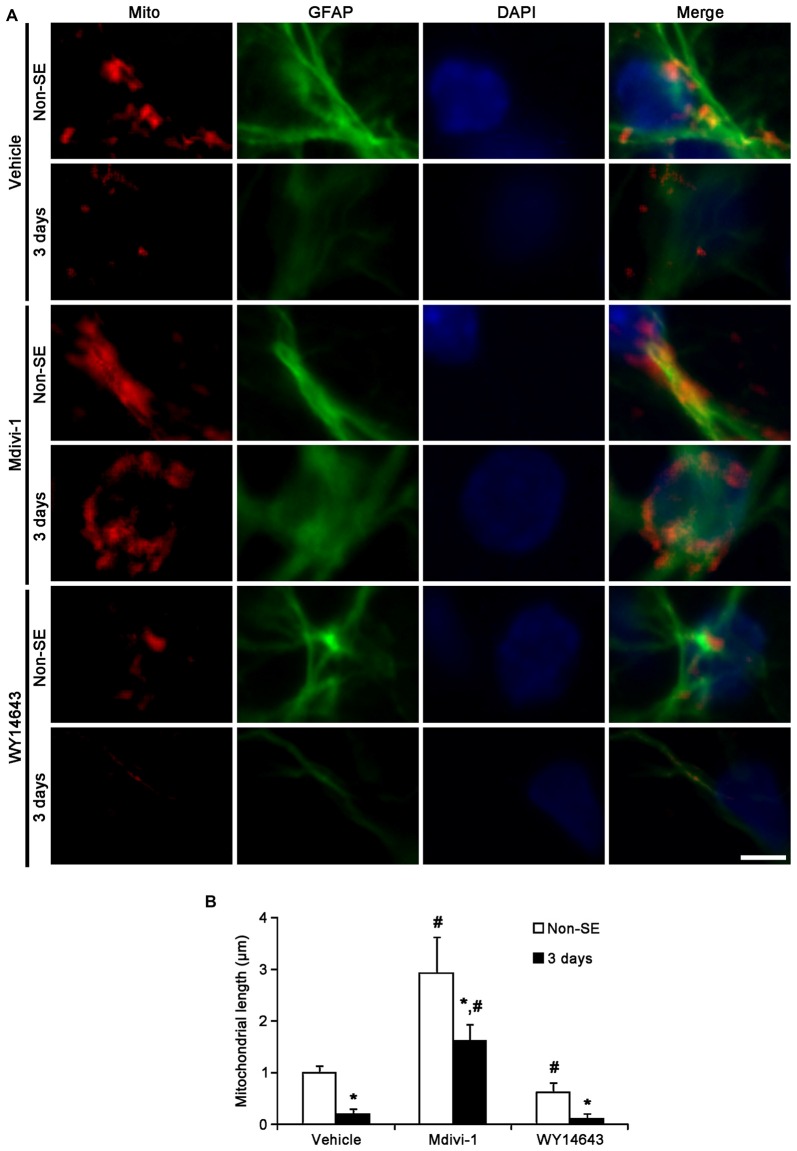
**Effects of Mdivi-1 and WY14643 on the mitochondrial length in astrocytes within the molecular layer of the dentate gyrus 3 days after SE. (A)** In non-SE animals, Mdivi-1 increases mitochondrial length, but WY14643 reduces it in astrocytes as compared to vehicle. Following SE, Mdivi-1 effectively attenuates reduction of mitochondrial length and induces hypertrophic and edematous changes in astrocytes without vacuolization. WY14643 does not affect reduction of mitochondrial length in astrocytes. Bar = 3.75 μm. **(B)** Quantitative values (mean ± SEM) of mitochondrial length in the molecular layer of the dentate gyrus (*n* = 7, respectively). *p < 0.05 vs. non-SE animals; ^#^*p* < 0.05 vs. vehicle.

**Figure 6:**
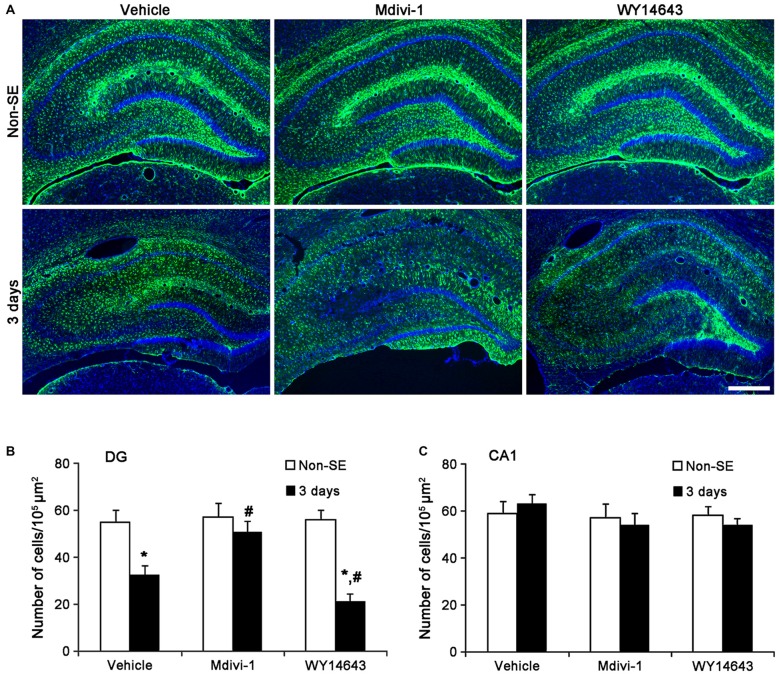
**Effects of Mdivi-1 and WY14643 on the regional specific astroglial death in response to SE. (A)** As compared to vehicle, Mdivi-1 effectively alleviates SE-induced astroglial death in the molecular layer of the dentate gyrus. In contrast, WY14643 aggravates SE-induced astroglial death in this region. Both Mdivi-1 and WY14643 did not affect the number of astrocytes in the CA1 region following SE. Bar = 6.25 μm.**(B)** Quantitative values (mean ± SEM) of the number of astrocytes in the molecular layer of the dentate gyrus (*n* = 7, respectively). *p < 0.05 vs. non-SE animals; ^#^*p* < 0.05 vs. vehicle. **(C)** Quantitative values (mean ± SEM) of the number of astrocytes in the CA1 region (*n* = 7, respectively).

In the molecular layer of the dentate gyrus, Mdivi-1 increased mitochondrial length (2.93 μm), but WY14643 reduced it (0.62 μm) in astrocytes of non-SE animals, as compared to vehicle (1 μm; *p* < 0.05 vs. vehicle; Figures 5A,B). Mdivi-1 effectively attenuated SE-induced reduction of mitochondrial length (1.62 μm) in astrocytes, as compared to vehicle (0.2 μm; *p* < 0.05 vs. vehicle; Figures 5A,B). In Mdivi-1 infused animals, furthermore, astrocytes had unevenly thick processes, and hypertrophic and edematous cell bodies without vacuolization. In contrast, WY14643 did not affect SE-induced reduction of mitochondrial length in astrocytes (0.1 μm) as compared to vehicle (0.2 μm; Figures 5A,B). Furthermore, Mdivi-1 effectively alleviated SE-induced astroglial death in the molecular layer of the dentate gyrus (*p* < 0.05 vs. vehicle, Figures 6A,B), although neither Mdivi-1 nor WY14643 induced astroglial death in non-SE animals (Figures 6A,B). In contrast, WY14643 aggravated SE-induced astroglial death in this region (*p* < 0.05 vs. vehicle, Figures 6A,B). These findings indicate that excessive mitochondrial fission may elicit SE-induced apoptotic astroglial death with the molecular layer of the dentate gyrus.

**Figure 7:**
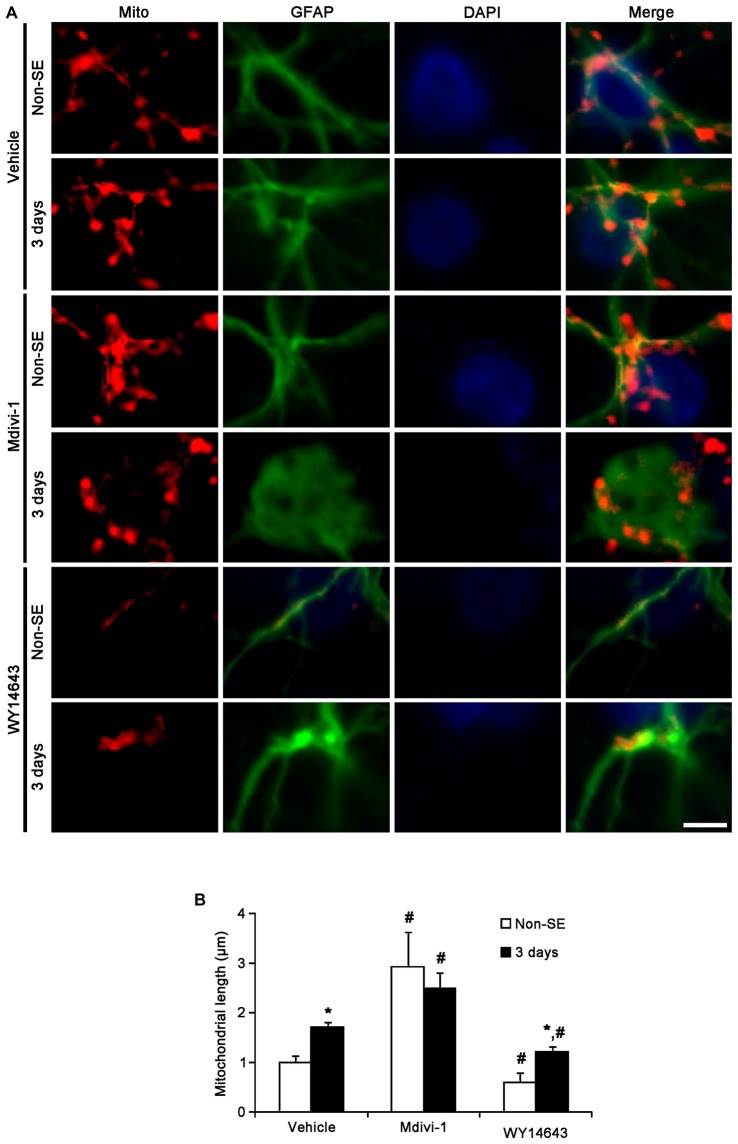
**Effects of Mdivi-1 and WY14643 on the mitochondrial length in CA1 astrocytes 3 days after SE. (A)** In non-SE animals, Mdivi-1 increases mitochondrial length, but WY14643 reduces it in astrocytes as compared to vehicle. Following SE, Mdivi-1 does not affect mitochondrial elongation, while WY14643 effectively inhibits it. CA1 astrocytes have round-shaped cell body, short blunt processes and vacuoles in the cytoplasm in Mdivi-1-treated animals, while CA1 astrocytes show typical reactive gliosis without vacuolization in vehicle- and WY14643-treated animals. Bar = 3.75 μm. **(B)** Quantitative values (mean ± SEM) of mitochondrial length in the molecular layer of the dentate gyrus (*n* = 7, respectively).*p < 0.05 vs. non-SE animals; ^#^*p* < 0.05 vs. vehicle.

**Figure 8:**
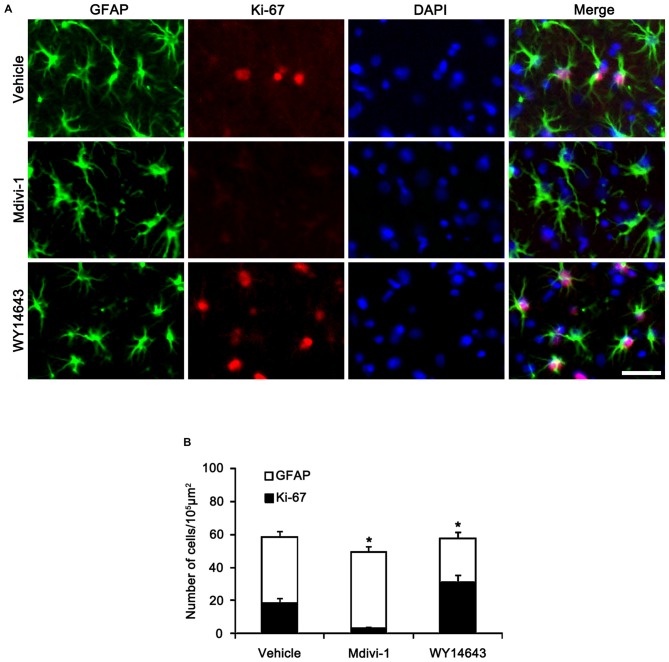
**Effects of Mdivi-1 and WY14643 on Ki-67 induction in CA1 astrocytes 3 days after SE. (A)** As compared to vehicle, Mdivi-1 infusion inhibits astroglial Ki-67 induction, while WY14643 infusion increases the number of Ki-67 positive astrocytes. Bar = 50 μm. **(B)** Quantitative values (mean ± SEM) of the fraction of Ki-67 positive astrocytes in total astrocytes (*n* = 7, respectively). *p < 0.05 vs. vehicle.

In the CA1 region, Mdivi-1 increased mitochondrial length (2.97 μm), but WY14643 decreased it in astrocytes of non-SE animals (0.59 μm; *p* < 0.05 vs. vehicle; Figures 7A,B). Although Mdivi-1 did not affect SE-induced mitochondrial elongation (2.49 μm), WY14643 effectively inhibited SE-induced elongation in astrocytes (1.21 μm) as compared to vehicle (1.71 μm; *p* < 0.05 vs. vehicle; Figures 7A,B). In vehicle- and WY14643-treated animals, astrocytes showed typical reactive gliosis (hypertrophy of cell bodies and processes) without vacuolization (*p* < 0.05 vs. vehicle; Figures 7A,B). In Mdivi-1-treated animals, astrocytes had round-shaped cell body, short blunt processes and vacuoles in the cytoplasm (*p* < 0.05 vs. vehicle; Figures 7A,B). Therefore, these findings demonstrate that abnormal mitochondrial elongation may accelerate clasmatodendrosis. Both Mdivi-1 and WY14643 did not affect the number of astrocytes in the CA1 region following SE (Figures 6A,C). However, Mdivi-1 infusion inhibited astroglial Ki-67 induction in this region (Figure 8; *p* < 0.05 vs. vehicle). In contrast, WY14643 infusion increased the number of Ki-67 positive astrocytes in the CA1 region (Figure 8; *p* < 0.05 vs. vehicle). These findings indicate that mitochondrial fission may be required for *in situ* proliferation of astrocytes in the CA1 region. Taken together, our findings suggest that the mitochondrial dynamics may play an important role in determining the cell death patterns as well as reactive gliosis of astrocytes in the CA1 region following SE.

### Regional Specific Astroglial DRP1 Phosphorylation in the Hippocampus

**Figure 9:**
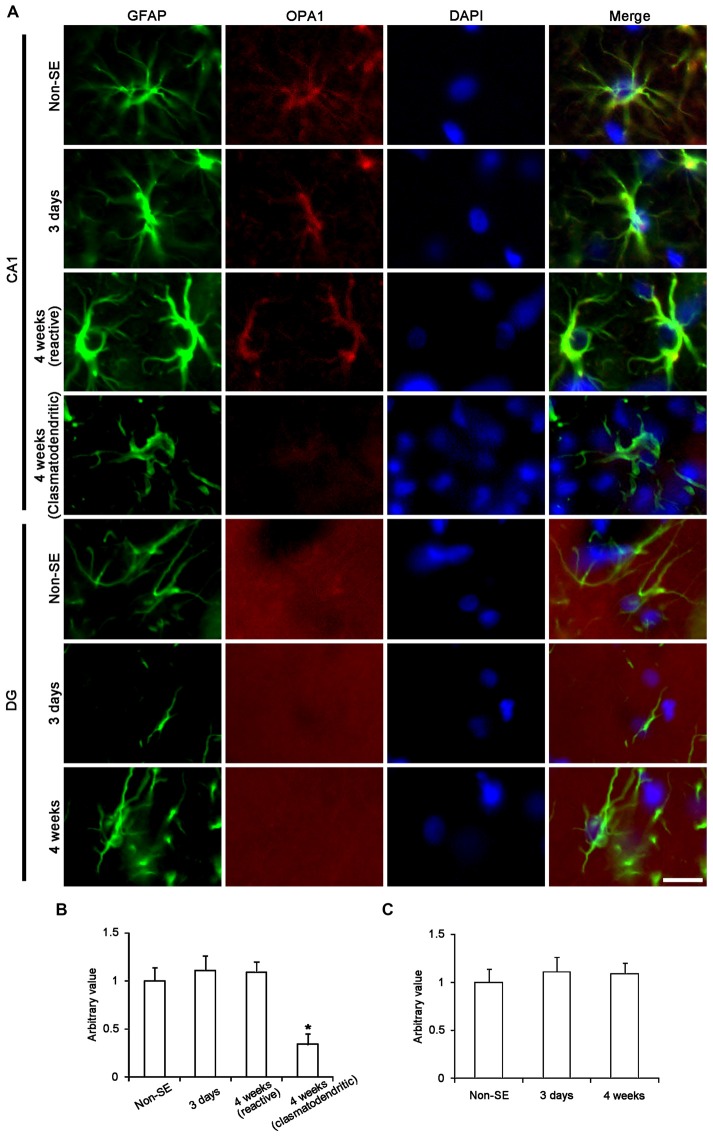
**Astroglial optic atrophy 1 (OPA1) expression in the hippocampus following SE. (A)** Double immunofluorescent images for GFAP and OPA1 following SE. In non-SE animals, OPA1 expression is clearly observed in CA1 astrocytes, while its expression is mainly detected in neuropils within the molecular layer of the dentate gyrus. As compared to non-SE animals, OPA1 expression is reduced in clasmatodendritic astrocytes, but not in reactive astrocytes. Bar = 6.25 μm. **(B,C)** Quantitative values (mean ± SEM) of OPA1 expression in the CA1 region **(B)** and the molecular layer of the dentate gyrus **(C)** (*n* = 7, respectively). *p < 0.05 vs. non-SE animals.

The remaining question is what kinds of mitochondrial dynamics-associated proteins are involved in SE-induced astroglial death. Since OPA1 is required for mitochondria fusion (Chen et al., 2003; Rambold et al., 2011), we investigated the alteration in OPA1 expression in astrocytes following SE. In non-SE animals, OPA1 expression was clearly observed in CA1 astrocytes, while its expression was predominantly detected in neuropils within the molecular layer of the dentate gyrus. SE did not affect OPA1 expression in astrocytes with both regions, whereas OPA1 expression was reduced in clasmatodendritic astrocytes (Figure 9).

**Figure 10:**
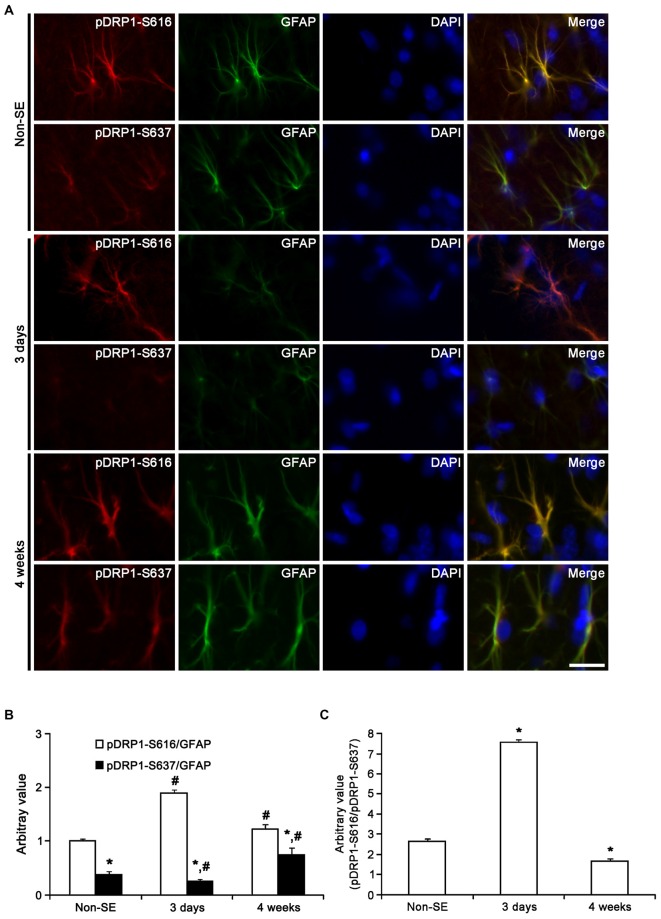
**Astroglial DRP1 phosphorylation in the molecular layer of the dentate gyrus following SE. (A)** Double immunofluorescent images for GFAP and pDRP1-S616/-S637 following SE. Three days after SE, pDRP1-S616 intensity is increased, while pDRP1-S637 intensity is reduced as compared to non-SE animals. Four weeks after SE, pDRP1-S616 intensity is unaltered, but pDRP1-S637 intensity is enhanced as compared to those observed in 3 days after SE. Bar = 12.5 μm. **(B)** Quantification of the ratio of pDRP1-S616/GFAP and pDRP1-S637/GFAP following SE (mean ± SEM, *n* = 7, respectively). *p < 0.05 vs. pDRP1-S616/GFAP ; ^#^*p* < 0.05 vs. non-SE animals. **(C)** Quantification of the ratio of pDRP1-S616/pDRP1-S637 following SE (mean ± SEM, *n* = 7, respectively). *p < 0.05 vs. non-SE animals.

**Figure 11:**
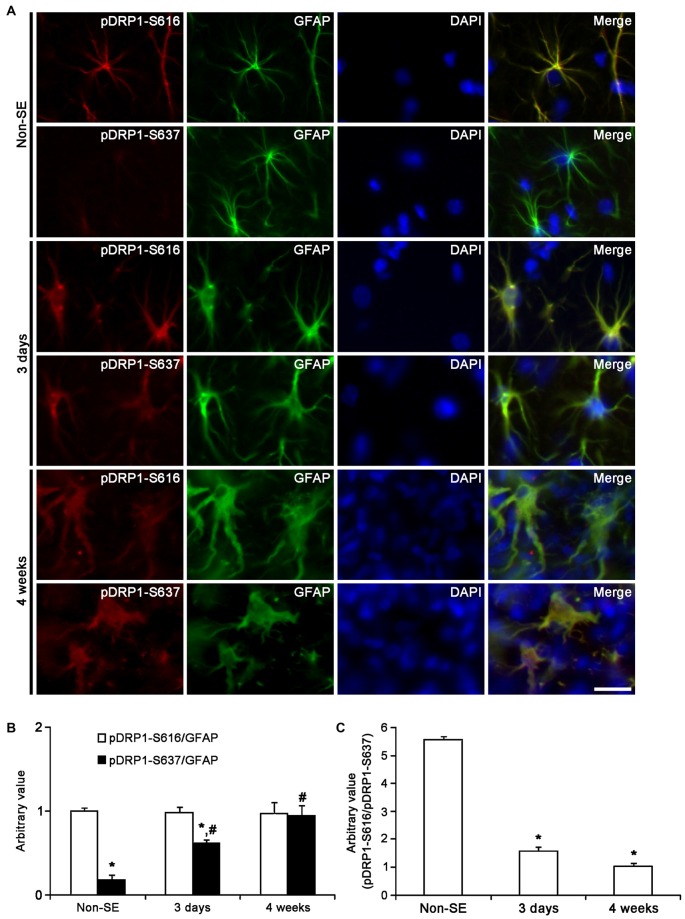
**Astroglial DRP1 phosphorylation in the CA1 region following SE. (A)** Double immunofluorescent images for GFAP and pDRP1-S616/-S637 following SE. Three days after SE, both pDRP1-S616 and pDRP1-S637 intensities are enhanced as compared to non-SE animals. Four weeks after SE, pDRP1-S616 intensity is unaltered, but pDRP1-S637 intensity is enhanced as compared to those observed in 3 days after SE. Bar = 12.5 μm. **(B)** Quantification of the ratio of pDRP1-S616/GFAP and pDRP1-S637/GFAP following SE (mean ± SEM, *n* = 7, respectively). *p < 0.05 vs. pDRP1-S616/GFAP; ^#^*p* < 0.05 vs. non-SE animals. **(C)** Quantification of the ratio of pDRP1-S616/pDRP1-S637 following SE (mean ± SEM, *n* = 7, respectively). *p < 0.05 vs. non-SE animals.

Unlike OPA1, DRP1 plays an important role in the modulation of mitochondrial fission (Smirnova et al., 2001), and involves various neurological diseases (DuBoff et al., 2012; Kim et al., 2014a). Mitochondrial dynamics are reversely regulated by DRP1 phosphorylation sites: DRP1-S616 phosphorylation activates mitochondrial fission, but DRP1-S637 phosphorylation inhibits fission (Kashatus et al., 2011; Wang et al., 2012). Therefore, DRP1 S616/S637 phosphorylation ratio is one of the indicatives for the capacity of DRP1-mediated mitochondrial dynamics (Kim et al., 2014a). To elucidate the relationship between mitochondrial dynamics and astroglial responses to SE, we analyzed the ratio of phospho-DRP1 (pDRP1) fluorescent intensities. In non-SE animals, the pDRP1-S616/pDRP1-S637 ratio in astrocytes was 2.63 within the molecular layer of the dentate gyrus (Figures 10A–C). In contrast, the pDRP1-S616/pDRP1-S637 ratio in astrocytes was 5.56 within the CA1 region (Figures 11A–C). These findings indicate that the potentials of mitochondrial fission in the CA1 region may be higher than that in the dentate gyrus.

Three days after SE, pDRP1-S616 intensity was increased to 1.89-fold of non-SE level, while pDRP1-S637 intensity was reduced to 0.66-fold of non-SE level in the molecular layer of the dentate gyrus (*p* < 0.05 vs. non-SE, Figures 10A,B). Therefore, pDRP1-S616/pDRP1-S637 ratio in astrocytes was increased to 7.56 (Figure 10C). In the CA1 region, pDRP1-S616 intensity was unaltered as compared to non-SE animals. However, pDRP1-S637/GFAP intensity was increased to 3.44-fold of non-SE level at 4 weeks after SE, respectively (*p* < 0.05 vs. non-SE, Figures 11A,B). Thus, pDRP1-S616/pDRP1-S637 ratio in astrocytes was reduced to 1.58 at 3 days after SE (*p* < 0.05 vs. non-SE, Figure 11C). These findings indicate that the potentials of DRP1-mediated mitochondrial fission in astrocytes may be increased in the molecular layer of the dentate gyrus, but be decreased in the CA1 region at 3 days after SE.

Four weeks after SE, pDRP1-S616/pDRP1-S637 ratio was declined to 1.66 in the molecular layer of the dentate gyrus (*p* < 0.05 vs. non-SE, Figure 10C), since pDRP1-S616 was 1.23-fold of non-SE level (*p* < 0.05 vs. non-SE, Figures 10A,B) and pDRP1-S637 intensity was enhanced to 1.95-fold of non-SE level (*p* < 0.05 vs. non-SE, Figures 10A,B). In the CA1 region, pDRP1-S637 intensity was increased to 5.22-fold of non-SE level at 4 weeks after SE, respectively (*p* < 0.05 vs. non-SE, Figures 11A,B). Thus, pDRP1-S616/pDRP1-S637 ratio in astrocytes was reduced to 1.03 at 4 weeks after SE, respectively (*p* < 0.05 vs. non-SE, Figure 11C). These findings suggest that SE may induce the prolonged reduction in the capacity of DRP1-mediated mitochondrial dynamics in CA1 astrocytes, unlike astrocytes in the molecular layer of the dentate gyrus. Therefore, it is likely that the distinctive DRP1 S616/S637 phosphorylation ratio in astrocytes may represent the differential properties of astrocytes, and may play an important role in the determination of SE-induced astroglial cell death patterns.

## Discussion

Astrocytes regulate the functions the blood–brain barrier, synaptic transmission and energy metabolism in the brain (Anderson and Swanson, 2000; Ransom et al., 2003). Following brain insults, reactive astrogliosis represents upregulated GFAP expression, astroglial hypertrophy, proliferation, and glial scar formation (Bordey and Sontheimer, 1998; Mathern et al., 1998). Therefore, astrocytes are considered to be resistant to various harmful stresses. However, Revuelta et al. (2005) reported astroglial death in the CA1 region after kainic acid administration. Astroglial apoptosis (Kang et al., 2006) and autophagic astroglial death (clasmatodendrosis; Kim et al., 2008, 2009; Ryu et al., 2011a) are also detected in the molecular layer of the dentate gyrus and the stratum radiatum of the CA1 region, respectively (Kim et al., 2008, 2009; Ryu et al., 2011a). Since SE induces hypotension, hyperpyrexia, hypoglycemia, acidosis and hypoxia (Turski et al., 1989; Walker et al., 2002), regional specific astroglial death would be simply interpreted as the results from impaired cellular metabolism induced by altered hemodynamics during SE. However, regional-specific astroglial death in response to SE is independent of hemodynamics, because astroglial responses in *ex vivo* study using brain slices are similar to those in *in vivo* model (Kim et al., 2014a). Therefore, it is noteworthy to elucidate the differential properties of astrocytes in the distinct regions in an effort to understand the role of astrocytes in various neurological diseases. Youle and Karbowski (2005) reported that excessive mitochondrial fission induces the execution of apoptosis. In addition, the interference of the mitochondrial fission inhibits apoptosis and evokes delayed cell death (Youle and Karbowski, 2005; Cassidy-Stone et al., 2008). Destabilization of DRP1 by mutant ubiquitin also enhances the mitochondrial fusion and the resistance to oxidative stress in astrocytes (Yim et al., 2014). Therefore, inhibition of mitochondrial fission would be considered beneficial for mitochondrial function. However, impaired mitochondrial fission also evokes a decrease in mitochondrial bioenergetics (Parone et al., 2008; DuBoff et al., 2012). Although mitochondrial elongation represents a mechanism for compensating a failure in energy production, elongated mitochondria can only transiently sustain cell metabolism (Rolland et al., 2013). In addition, DRP1 deletion impairs mitochondrial functions due to oxidative stress that leads to further aggravation of mitochondrial damage via elevated synthesis of reactive oxygen species (Parone et al., 2008; Kageyama et al., 2012), which can activate autophagic cell death (Chen et al., 2007; Lin et al., 2014). In the present study, SE induced mitochondrial elongation and clasmatodendritic astroglial degeneration by reduction in DRP1 S616/S637 phosphorylation ratio in CA1 astrocytes. In contrast to the CA1 region, SE resulted in mitochondrial fission and apoptotic astroglial death by increased DRP1 S616/S637 phosphorylation ratio in the dentate gyrus. As compared to non-SE animals, OPA1 expression in astrocytes was unaltered following SE, although its expression was declined in clasmatodendritic (dying) astrocytes. Furthermore, Mdivi-1 significantly deteriorated mitochondrial elongation and clasmatodendrosis-like event in CA1 astrocytes. Mdivi-1 also prevented SE-induced apoptotic astroglial death in the dentate gyrus, while WY14643 aggravated it. These findings indicate that blockade of excessive mitochondrial fission may prevent astroglial apoptosis, but the sustained inhibition may result in astroglial dysfunction and ultimately autophagic death in astrocytes. Therefore, our data propose that DRP1-mediated mitochondrial dynamics may be a double-edge sword in SE-induced astroglial death.

Clasmatodendrosis is an irreversible astroglial damage reported by Alzheimer in 1910, which includes extensive swelling and vacuolization of cell bodies and disintegrated and beaded processes, and termed “clasmatodendrosis” by Cajal. In addition, the degeneration of CA1 astrocytes is observed in various models for neurological diseases (Tomimoto et al., 1997; Deloncle et al., 2001; Sugawara et al., 2002; Revuelta et al., 2005; Kim et al., 2008), which is characterized by TUNEL negative hypertrophy and vacuolization. This vacuolized astroglial degeneration would be considered as coagulative necrotic changes (Sugawara et al., 2002). However, we discovered that vacuoles in clasmatodendritic astrocytes showed LAMP-1 immunoreactivity, and both LC3-II and Beclin-1 expression were detected in most of clasmatodendritic astrocytes (Ryu et al., 2011a). Therefore, we have reported that clasmatodendrosis may be autophagic astroglial death. Although the exact mechanism has been unknown, clasmatodendrosis is exclusively observed in the CA1 region following ischemic insults or excitotoxic injury (Dihné et al., 2001; Sugawara et al., 2002). Thus, some investigators speculate that clasmatodendrosis is relevant to astroglial dysfunction due to energy failure and acidosis coupled to mitochondrial inhibition (Friede and van Houten, 1961; Kraig and Chesler, 1990; Hulse et al., 2001). Based on these previous studies, our findings provide the possibility that clasmatodendrosis may be closely related to mitochondrial dysfunction due to prolonged impairment of mitochondrial fission.

It is well known that the vulnerability of neurons to insults is not uniform in the hippocampus, but is heterogeneous among the various brain regions. Briefly, dentate granule cells are remarkably resistant to neuronal damage caused by most insults. Conversely, the CA1 and CA3 neurons as well as hilar neurons are extremely vulnerable to various harmful stimuli (Greene et al., 2009). However, the reason or mechanism of these differential vulnerability of hippocampal neurons is still unknown. Aforementioned, increasing evidence supports that SE leads to devastating astroglial death that is characterized by regional-specific patterns, since Schmidt-Kastner and Ingvar (1994, 1996) described regional-specific astroglial damage in the brain after SE. Similar to the case of hippocampal neurons, the molecular events underlying the occurrence of regional-specific astroglial death are unclear. At first, we hypothesized that the differential astrocyte physiology between CA1 and dentate gyrus would be most likely because of the distinctive hemodynamic characteristics between the dentate gyrus and the hippocampus *in vivo*. However, astroglial response to pilocarpine in *ex vivo* model is similar to that in *in vivo* model (Kim et al., 2014a). These findings indicate that the regional-specific pattern of astroglial responses may be hemodynamic-independent phenomena. Therefore, we conclude that regional-specific astroglial responses to SE may be consequences from the differential astroglial profiles *per se*. In the present study, the morphology of mitochondria was conserved between CA1 astrocytes and astrocytes within the molecular layer of the dentate gyrus. However, DRP1 S616/S637 phosphorylation ratio in CA1 astrocytes was higher than in the molecular layer of the dentate gyrus. These findings indicate the higher potentials of mitochondrial fission and DRP1 activity in CA1 astrocytes than in astrocytes within the dentate gyrus. Interestingly, the present data also demonstrates that SE elongated mitochondrial length in CA1 astrocytes via increase in pDRP1-S637 level, while it increased mitochondrial fission due to enhancement of pDRP1-S616, and concomitantly resulted in astroglial death in the dentate gyrus. Therefore, it is likely that the distinctive DRP1 S616/S637 phosphorylation ratio in naïve astrocytes may represent the differential properties of astrocytes, and may play an important role in the proper maintenance of mitochondrial dynamics in response to mitochondrial division-inducing events.

The present data cannot directly address the mechanism by which SE affects mitochondrial dynamics in astrocytes. Horn et al. (2011) reported that DRP1 activity drops when cells enter G1 phase, and subsequently gradually increases after G1/S arrest. Furthermore, mitotic mitochondrial fission depends on DRP1 S616 phosphorylation by cyclin B/cyclin-dependent kinase 1 (Kashatus et al., 2011). In the present study, unexpectedly, Mdivi-1 inhibited astroglial Ki-67 induction, whereas WY14643 increased the number of Ki-67 positive astrocytes following SE. Therefore, it is presumable that mitochondrial dynamics and cell-cycle related events may be reciprocally regulated by each other during the development of reactive astrogliosis induced by SE, which is unknown at present. Alternatively, glutamate excitotoxicity may be involved in dysfunction of mitochondrial dynamics in astrocytes (Szydlowska et al., 2006; Ju et al., 2015). Indeed, blockade of glutamate excitotoxicity promotes astroglial viability (Lee et al., 2010). Therefore, it is likely that the distinct susceptibility to glutamate or glutamate concentration may lead to the differential patterns of imbalance of mitochondrial dynamics. Further studies are needed to elucidate the exact mechanism of regulation of astroglial DRP1 activity induced by SE.

In conclusion, the present findings indicate that naïve astrocytes display unique patterns of DRP1 phosphorylation in the different hippocampal regions, which is relevant to the distinct astroglial responses to SE. In addition, our findings represent that enhanced pDRP1-S637 promoted autophagic astroglial death, while increased pDRP-S616 could induce apoptotic astroglial death. Therefore, the regulation of DRP1 phosphorylation may be one of the important factors to determine the patterns of astroglial death induced by SE.

## Author Contributions

J-EK designed and supervised the project. J-EK designed and performed the experiments described in the manuscript with A-RK, H-WH and S-JM. H-WH, A-RK and J-EK analyzed the data. J-EK wrote the manuscript.

## Conflict of Interest Statement

The authors declare that the research was conducted in the absence of any commercial or financial relationships that could be construed as a potential conflict of interest.

## References

[B1] Alaimo A. Gorojod R. M. Beauquis J. Muñoz M. J. Saravia F. Kotler M. L. (2014). Deregulation of mitochondria-shaping proteins Opa-1 and Drp-1 in manganese-induced apoptosis. PLoS One 9:e91848. 10.1371/journal.pone.0091848 24632637 PMC3954806

[B2] Anderson C. M. Swanson R. A. (2000). Astrocyte glutamate transport: review of properties, regulation and physiological functions. Glia 32, 1–14. 10.1002/1098-1136(200010)32:1<1::aid-glia10>3.3.co;2-n 10975906

[B3] Bach D. Pich S. Soriano F. X. Vega N. Baumgartner B. Oriola J.. (2003). Mitofusin-2 determines mitochondrial network architecture and mitochondrial metabolism. A novel regulatory mechanism altered in obesity. J. Biol. Chem. 278, 17190–17197. 10.1074/jbc.m212754200 12598526

[B4] Benard G. Rossignol R. (2008). Ultrastructure of the mitochondrion and its bearing on function and bioenergetics. Antioxid. Redox Signal. 10, 1313–1342. 10.1089/ars.2007.2000 18435594

[B5] Birsa N. Norkett R. Higgs N. Lopez-Domenech G. Kittler J. T. (2013). Mitochondrial trafficking in neurons and the role of the miro family of GTPase proteins. Biochem. Soc. Trans. 41, 1525–1531. 10.1042/BST20130234 24256248

[B6] Bordey A. Sontheimer H. (1998). Properties of human glial cells associated with epileptic seizure foci. Epilepsy Res. 32, 286–303. 10.1016/s0920-1211(98)00059-x 9761328

[B7] Borges K. McDermott D. Irier H. Smith Y. Dingledine R. (2006). Degeneration and proliferation of astrocytes in the mouse dentate gyrus after pilocarpine-induced status epilepticus. Exp. Neurol. 201, 416–427. 10.1016/j.expneurol.2006.04.031 16793040 PMC4090707

[B8] Campello S. Scorrano L. (2010). Mitochondrial shape changes: orchestrating cell pathophysiology. EMBO Rep. 11, 678–684. 10.1038/embor.2010.115 20725092 PMC2933866

[B9] Cassidy-Stone A. Chipuk J. E. Ingerman E. Song C. Yoo C. Kuwana T.. (2008). Chemical inhibition of the mitochondrial division dynamin reveals its role in Bax/Bak-dependent mitochondrial outer membrane permeabilization. Dev. Cell 14, 193–204. 10.1016/j.devcel.2007.11.019 18267088 PMC2267902

[B11] Chen H. Chan D. C. (2005). Emerging functions of mammalian mitochondrial fusion and fission. Hum. Mol. Genet. 14, R283–R289. 10.1093/hmg/ddi270 16244327

[B10] Chen H. Chan D. C. (2009). Mitochondrial dynamics-fusion, fission, movement and mitophagy-in neurodegenerative diseases. Hum. Mol. Genet. 18, R169–R176. 10.1093/hmg/ddp326 19808793 PMC2758711

[B12] Chen H. Chomyn A. Chan D. C. (2005). Disruption of fusion results in mitochondrial heterogeneity and dysfunction. J. Biol. Chem. 280, 26185–26192. 10.1074/jbc.m503062200 15899901

[B13] Chen H. Detmer S. A. Ewald A. J. Griffin E. E. Fraser S. E. Chan D. C. (2003). Mitofusins Mfn1 and Mfn2 coordinately regulate mitochondrial fusion and are essential for embryonic development. J. Cell Biol. 160, 189–200. 10.1083/jcb.200211046 12527753 PMC2172648

[B14] Chen Y. McMillan-Ward E. Kong J. Israels S. J. Gibson S. B. (2007). Mitochondrial electron-transport-chain inhibitors of complexes I and II induce autophagic cell death mediated by reactive oxygen species. J. Cell Sci. 120, 4155–4166. 10.1242/jcs.011163 18032788

[B15] Cheung E. C. McBride H. M. Slack R. S. (2007). Mitochondrial dynamics in the regulation of neuronal cell death. Apoptosis 12, 979–992. 10.1007/s10495-007-0745-5 17453163

[B16] Cipolat S. Martins de Brito O. Dal Zilio B. Scorrano L. (2004). OPA1 requires mitofusin 1 to promote mitochondrial fusion. Proc. Natl. Acad. Sci. U S A 101, 15927–15932. 10.1073/pnas.0407043101 15509649 PMC528769

[B17] Deloncle R. Huguet F. Fernandez B. Quellard N. Babin P. Guillard O. (2001). Ultrastructural study of rat hippocampus after chronic administration of aluminum L-glutamate: an acceleration of the aging process. Exp. Gerontol. 36, 231–244. 10.1016/s0531-5565(00)00214-x 11226739

[B18] Detmer S. A. Chan D. C. (2007). Functions and dysfunctions of mitochondrial dynamics. Nat. Rev. Mol. Cell Biol. 8, 870–879. 10.1038/nrm2275 17928812

[B19] Dihné M. Block F. Korr H. Töpper R. (2001). Time course of glial proliferation and glial apoptosis following excitotoxic CNS injury. Brain Res. 902, 178–189. 10.1016/s0006-8993(01)02378-2 11384611

[B20] DuBoff B. Götz J. Feany M. B. (2012). Tau promotes neurodegeneration via DRP1 mislocalization *in vivo*. Neuron 75, 618–632. 10.1016/j.neuron.2012.06.026 22920254 PMC3428596

[B21] Friede R. L. van Houten W. H. (1961). Relations between postmortem alterations and glycolytic metabolism in the brain. Exp. Neurol. 4, 197–204. 10.1016/0014-4886(61)90041-3 13895193

[B22] Greene J. G. Borges K. Dingledine R. (2009). Quantitative transcriptional neuroanatomy of the rat hippocampus: evidence for wide-ranging, pathway-specific heterogeneity among three principal cell layers. Hippocampus 19, 253–264. 10.1002/hipo.20502 18830999 PMC2649995

[B23] Gualtieri F. Curia G. Marinelli C. Biagini G. (2012). Increased perivascular laminin predicts damage to astrocytes in CA3 and piriform cortex following chemoconvulsive treatments. Neuroscience 218, 278–294. 10.1016/j.neuroscience.2012.05.018 22609936

[B24] Horn S. R. Thomenius M. J. Johnson E. S. Freel C. D. Wu J. Q. Coloff J. L.. (2011). Regulation of mitochondrial morphology by APC/CCdh1-mediated control of Drp1 stability. Mol. Biol. Cell 22, 1207–1216. 10.1091/mbc.e10-07-0567 21325626 PMC3078078

[B25] Hulse R. E. Winterfield J. Kunkler P. E. Kraig R. P. (2001). Astrocytic clasmatodendrosis in hippocampal organ culture. Glia 33, 169–179. 10.1002/1098-1136(200102)33:23.0.CO;2-B 11180514 PMC2807126

[B26] Ingvar M. Schmidt-Kastner R. Meller D. (1994). Immunohistochemical markers for neurons and astrocytes show pan-necrosis following infusion of high-dose NMDA into rat cortex. Exp. Neurol. 128, 249–259. 10.1006/exnr.1994.1134 8076669

[B27] Jahani-Asl A. Cheung E. C. Neuspiel M. MacLaurin J. G. Fortin A. Park D. S.. (2007). Mitofusin 2 protects cerebellar granule neurons against injury-induced cell death. J. Biol. Chem. 282, 23788–23798. 10.1074/jbc.m703812200 17537722

[B28] Ju W. K. Kim K. Y. Noh Y. H. Hoshijima M. Lukas T. J. Ellisman M. H.. (2015). Increased mitochondrial fission and volume density by blocking glutamate excitotoxicity protect glaucomatous optic nerve head astrocytes. Glia 63, 736–753. 10.1002/glia.22781 25557093 PMC4373968

[B29] Kageyama Y. Zhang Z. Roda R. Fukaya M. Wakabayashi J. Wakabayashi N.. (2012). Mitochondrial division ensures the survival of postmitotic neurons by suppressing oxidative damage. J. Cell Biol. 197, 535–551. 10.1083/jcb.201110034 22564413 PMC3352955

[B30] Kang T. C. Kim D. S. Kwak S. E. Kim J. E. Won M. H. Kim D. W.. (2006). Epileptogenic roles of astroglial death and regeneration in the dentate gyrus of experimental temporal lobe epilepsy. Glia 54, 258–271. 10.1002/glia.20380 16845674

[B31] Kashatus D. F. Lim K. H. Brady D. C. Pershing N. L. Cox A. D. Counter C. M. (2011). RALA and RALBP1 regulate mitochondrial fission at mitosis. Nat. Cell Biol. 13, 1108–1115. 10.1038/ncb2310 21822277 PMC3167028

[B33] Kim J. E. Kim Y. J. Kim J. Y. Kang T. C. (2014a). PARP1 activation/expression modulates regional-specific neuronal and glial responses to seizure in a hemodynamic-independent manner. Cell Death Dis. 5:e1362. 10.1038/cddis.2014.331 25101675 PMC4454306

[B35] Kim J. E. Ryu H. J. Kim M. J. Kang T. C. (2014b). LIM kinase-2 induces programmed necrotic neuronal death via dysfunction of DRP1-mediated mitochondrial fission. Cell Death Differ. 21, 1036–1049. 10.1038/cdd.2014.17 24561342 PMC4207472

[B32] Kim D. S. Kim J. E. Kwak S. E. Choi K. C. Kim D. W. Kwon O. S.. (2008). Spatiotemporal characteristics of astroglial death in the rat hippocampo-entorhinal complex following pilocarpine-induced status epilepticus. J. Comp. Neurol. 511, 581–598. 10.1002/cne.21851 18853423

[B34] Kim J. E. Ryu H. J. Kang T. C. (2013). Status epilepticus induces vasogenic edema via tumor necrosis factor-α/endothelin-1-mediated two different pathways. PLoS One 8:e74458. 10.1371/journal.pone.0074458 24040253 PMC3764062

[B36] Kim J. E. Ryu H. J. Kim M. J. Kim D. W. Kwon O. S. Choi S. Y.. (2010a). Pyridoxal-5′-phosphate phosphatase/chronophin induces astroglial apoptosis via actin-depolymerizing factor/cofilin system in the rat brain following status epilepticus. Glia 58, 1937–1948. 10.1002/glia.21063 20737471

[B39] Kim J. E. Yeo S. I. Ryu H. J. Kim M. J. Kim D. S. Jo S. M.. (2010b). Astroglial loss and edema formation in the rat piriform cortex and hippocampus following pilocarpine-induced status epilepticus. J. Comp. Neurol. 518, 4612–4628. 10.1002/cne.22482 20886625

[B37] Kim J. E. Ryu H. J. Yeo S. I. Kang T. C. (2011). P2X7 receptor differentially modulates astroglial apoptosis and clasmatodendrosis in the rat brain following status epilepticus. Hippocampus 21, 1318–1333. 10.1002/hipo.20850 20848604

[B38] Kim J. E. Ryu H. J. Yeo S. I. Seo C. H. Lee B. C. Choi I. G.. (2009). Differential expressions of aquaporin subtypes in astroglia in the hippocampus of chronic epileptic rats. Neuroscience 163, 781–789. 10.1016/j.neuroscience.2009.07.028 19619613

[B40] Kraig R. P. Chesler M. (1990). Astrocytic acidosis in hyperglycemic and complete ischemia. J. Cereb. Blood Flow Metab. 10, 104–114. 10.1038/jcbfm.1990.13 2298827 PMC3047406

[B41] Lee M. C. Ting K. K. Adams S. Brew B. J. Chung R. Guillemin G. J. (2010). Characterisation of the expression of NMDA receptors in human astrocytes. PLoS One 5:e14123. 10.1371/journal.pone.0014123 21152063 PMC2994931

[B42] Li Z. Okamoto K. Hayashi Y. Sheng M. (2004). The importance of dendritic mitochondria in the morphogenesis and plasticity of spines and synapses. Cell 119, 873–887. 10.1016/j.cell.2004.11.003 15607982

[B43] Liesa M. Palacín M. Zorzano A. (2009). Mitochondrial dynamics in mammalian health and disease. Physiol. Rev. 89, 799–845. 10.1152/physrev.00030.2008 19584314

[B44] Lin C. J. Chen T. H. Yang L. Y. Shih C. M. (2014). Resveratrol protects astrocytes against traumatic brain injury through inhibiting apoptotic and autophagic cell death. Cell Death Dis. 5:e1147. 10.1038/cddis.2014.123 24675465 PMC3973229

[B45] Lundgren B. Bergstrand A. Karlsson K. DePierre J. W. (1990). Effects of dietary treatment with clofibrate, nafenopin or WY-14.643 on mitochondria and DNA in mouse liver. Biochim. Biophys. Acta 1035, 132–138. 10.1016/0304-4165(90)90107-8 2393663

[B46] MacAskill A. F. Atkin T. A. Kittler J. T. (2010). Mitochondrial trafficking and the provision of energy and calcium buffering at excitatory synapses. Eur. J. Neurosci. 32, 231–240. 10.1111/j.1460-9568.2010.07345.x 20946113

[B47] Mathern G. W. Pretorius J. K. Kornblum H. I. Mendoza D. Lozada A. Leite J. P.. (1998). Altered hippocampal kainate-receptor mRNA levels in temporal lobe epilepsy patients. Neurobiol. Dis. 5, 151–176. 10.1006/nbdi.1998.0200 9848088

[B48] Olichon A. Baricault L. Gas N. Guillou E. Valette A. Belenguer P.. (2003). Loss of OPA1 perturbates the mitochondrial inner membrane structure and integrity, leading to cytochrome c release and apoptosis. J. Biol. Chem. 278, 7743–7746. 10.1074/jbc.c200677200 12509422

[B49] Parone P. A. Da Cruz S. Tondera D. Mattenberger Y. James D. I. Maechler P.. (2008). Preventing mitochondrial fission impairs mitochondrial function and leads to loss of mitochondrial DNA. PLoS One 3:e3257. 10.1371/journal.pone.0003257 18806874 PMC2532749

[B50] Penfield W. (1928). “Neuroglia and microglia—the interstitial tissue of the central nervous system,” in Special Cytology, the Form and Function of the Cell in Health and Disease, ed. Cowdry E. V. (New York, NY: Hoeber), 1033–1068.

[B51] Rambold A. S. Kostelecky B. Elia N. Lippincott-Schwartz J. (2011). Tubular network formation protects mitochondria from autophagosomal degradation during nutrient starvation. Proc. Natl. Acad. Sci. U S A 108, 10190–10195. 10.1073/pnas.1107402108 21646527 PMC3121813

[B52] Ransom B. Behar T. Nedergaard M. (2003). New roles for astrocytes (stars at last). Trends Neurosci. 26, 520–522. 10.1016/j.tins.2003.08.006 14522143

[B53] Revuelta M. Castaño A. Machado A. Cano J. Venero J. L. (2005). Kainate-induced zinc translocation from presynaptic terminals causes neuronal and astroglial cell death and mRNA loss of BDNF receptors in the hippocampal formation and amygdala. J. Neurosci. Res. 82, 184–195. 10.1002/jnr.20632 16175575

[B54] Rintoul G. L. Reynolds I. J. (2010). Mitochondrial trafficking and morphology in neuronal injury. Biochim. Biophys. Acta 1802, 143–150. 10.1016/j.bbadis.2009.09.005 19747973

[B55] Rolland S. G. Motori E. Memar N. Hench J. Frank S. Winklhofer K. F.. (2013). Impaired complex IV activity in response to loss of LRPPRC function can be compensated by mitochondrial hyperfusion. Proc. Natl. Acad. Sci. U S A 110, E2967–E2976. 10.1073/pnas.1303872110 23878239 PMC3740885

[B56] Ryu H. J. Kim J. E. Yeo S. I. Kang T. C. (2011a). p65/RelA-Ser529 NF-κB subunit phosphorylation induces autophagic astroglial death (Clasmatodendrosis) following status epilepticus. Cell Mol. Neurobiol. 31, 1071–1078. 10.1007/s10571-011-9706-1 21598036 PMC11498587

[B57] Ryu H. J. Kim J. E. Yeo S. I. Kim D. W. Kwon O. S. Choi S. Y.. (2011b). F-actin depolymerization accelerates clasmatodendrosis via activation of lysosome-derived autophagic astroglial death. Brain Res. Bull. 85, 368–373. 10.1016/j.brainresbull.2011.05.007 21624438

[B58] Schmidt-Kastner R. Ingvar M. (1994). Loss of immunoreactivity for glial fibrillary acidic protein (GFAP) in astrocytes as a marker for profound tissue damage in substantia nigra and basal cortical areas after status epilepticus induced by pilocarpine in rat. Glia 12, 165–172. 10.1002/glia.440120302 7851985

[B59] Schmidt-Kastner R. Ingvar M. (1996). Laminar damage of neurons and astrocytes in neocortex and hippocampus of rat after long-lasting status epilepticus induced by pilocarpine. Epilepsy Res. Suppl. 12, 309–316. 9302530

[B60] Sheng Z. H. Cai Q. (2012). Mitochondrial transport in neurons: impact on synaptic homeostasis and neurodegeneration. Nat. Rev. Neurosci. 13, 77–93. 10.1038/nrn3156 22218207 PMC4962561

[B61] Smirnova E. Griparic L. Shurland D. L. van der Bliek A. M. (2001). Dynamin-related protein Drp1 is required for mitochondrial division in mammalian cells. Mol. Biol. Cell 12, 2245–2256. 10.1091/mbc.12.8.2245 11514614 PMC58592

[B62] Sugawara T. Lewén A. Noshita N. Gasche Y. Chan P. H. (2002). Effects of global ischemia duration on neuronal, astroglial, oligodendroglial and microglial reactions in the vulnerable hippocampal CA1 subregion in rats. J. Neurotrauma 19, 85–98. 10.1089/089771502753460268 11852981

[B63] Sung J. Y. Engmann O. Teylan M. A. Nairn A. C. Greengard P. Kim Y. (2008). WAVE1 controls neuronal activity-induced mitochondrial distribution in dendritic spines. Proc. Natl. Acad. Sci. U S A 105, 3112–3116. 10.1073/pnas.0712180105 18287015 PMC2268593

[B64] Szydlowska K. Zawadzka M. Kaminska B. (2006). Neuroprotectant FK506 inhibits glutamate-induced apoptosis of astrocytes *in vitro* and *in vivo*. J. Neurochem. 99, 965–975. 10.1111/j.1471-4159.2006.04136.x 17076660

[B65] Taguchi N. Ishihara N. Jofuku A. Oka T. Mihara K. (2007). Mitotic phosphorylation of dynamin-related GTPase Drp1 participates in mitochondrial fission. J. Biol. Chem. 282, 11521–11529. 10.1074/jbc.m607279200 17301055

[B66] Tomimoto H. Akiguchi I. Wakita H. Suenaga T. Nakamura S. Kimura J. (1997). Regressive changes of astroglia in white matter lesions in cerebrovascular disease and Alzheimer’s disease patients. Acta Neuropathol. 94, 146–152. 10.1007/s004010050686 9255389

[B67] Turski L. Ikonomidou C. Turski W. A. Bortolotto Z. A. Cavalheiro E. A. (1989). Review: cholinergic mechanisms and epileptogenesis. The seizures induced by pilocarpine: a novel experimental model of intractable epilepsy. Synapse 3, 154–171. 10.1002/syn.890030207 2648633

[B68] Walker M. C. White H. S. Sander J. W. (2002). Disease modification in partial epilepsy. Brain 125, 1937–1950. 10.1093/brain/awf203 12183340

[B69] Wang Z. Jiang H. Chen S. Du F. Wang X. (2012). The mitochondrial phosphatase PGAM5 functions at the convergence point of multiple necrotic death pathways. Cell 148, 228–243. 10.1016/j.cell.2011.11.030 22265414

[B70] Yim N. Ryu S. W. Han E. C. Yoon J. Choi K. Choi C. (2014). Mutant ubiquitin UBB+1 inducesmitochondrial fusion by destabilizing mitochondrial fission-specific proteins and confers resistance to oxidative stress-induced cell death in astrocytic cells. PLoS One 9:e99937. 10.1371/journal.pone.0099937 24941066 PMC4062464

[B71] Youle R. J. Karbowski M. (2005). Mitochondrial fission in apoptosis. Nat. Rev. Mol. Cell Biol. 6, 657–663. 10.1038/nrm1697 16025099

[B72] Zolezzi J. M. Silva-Alvarez C. Ordenes D. Godoy J. A. Carvajal F. J. Santos M. J.. (2013). Peroxisome proliferator-activated receptor (PPAR) γ and PPARα agonists modulate mitochondrial fusion-fission dynamics: relevance to reactive oxygen species (ROS)-related neurodegenerative disorders? PLoS One 8:e64019. 10.1371/journal.pone.0064019 23675519 PMC3652852

